# Ethical problems in pediatrics: what does the setting of care and education show us?

**DOI:** 10.1186/1472-6939-13-2

**Published:** 2012-03-16

**Authors:** Jucélia Maria Guedert, Suely Grosseman

**Affiliations:** 1 Postgraduate Program in Medical Sciences, Federal University of Santa Catarina, Florianópolis, Brazil

## Abstract

**Background:**

Pediatrics ethics education should enhance medical students' skills to deal with ethical problems that may arise in the different settings of care. This study aimed to analyze the ethical problems experienced by physicians who have medical education and pediatric care responsibilities, and if those problems are associated to their workplace, medical specialty and area of clinical practice.

**Methods:**

A self-applied semi-structured questionnaire was answered by 88 physicians with teaching and pediatric care responsibilities. Content analysis was performed to analyze the qualitative data. Poisson regression was used to explore the association of the categories of ethical problems reported with workplace and professional specialty and activity.

**Results:**

210 ethical problems were reported, grouped into five areas: physician-patient relationship, end-of-life care, health professional conducts, socioeconomic issues and health policies, and pediatric teaching. Doctors who worked in hospitals as well as general and subspecialist pediatricians reported fewer ethical problems related to socioeconomic issues and health policies than those who worked in Basic Health Units and who were family doctors.

**Conclusions:**

Some ethical problems are specific to certain settings: those related to end-of-life care are more frequent in the hospital settings and those associated with socioeconomic issues and public health policies are more frequent in Basic Health Units. Other problems are present in all the setting of pediatric care and learning and include ethical problems related to physician-patient relationship, health professional conducts and the pediatric education process. These findings should be taken into consideration when planning the teaching of ethics in pediatrics.

**Trial registration:**

This research article didn't reports the results of a controlled health care intervention. The study project was approved by the Institutional Ethical Review Committee (Report CEP-HIJG 032/2008).

## Background

Many facets of contemporary society are challenging the health care arena and demand constant reflection about the best professional attitudes to be taken in a diversity of circumstances. In this context, the current way of teaching ethics in medicine has been changed and transcends the traditional model of deontological ethics. The moral education and the rescue and cultivation of qualities and attitudes of a virtuous person required for good medical practice (virtue-based ethics) has been a pressing need [1-4].

The Brazilian Constitution [5] (Article 227) and the Child and Adolescent Statute [6] (Law 8069/90), which domestically put into force the International Convention on the Rights of the Child and the Universal Declaration of the Rights of the Child, establish Brazil's policy of full protection for children as law. These legal instruments conceive of children as citizens who have full rights and who are subject to protective priority because of their physical, psychological and moral vulnerability. However, despite the legislative and social advances of recent decades, Brazil still has significant work to do to advance the care and protection of children and adolescents, especially regarding access to quality education and the fight against malnutrition, child labor, abuse, neglect and all forms of violence against children.

Pediatrics, an area with complex interpersonal interactions and heavily influenced by emotions, has the potential to give rise to situations involving ethical problems. Entities engaged in medical education have developed and released key documents on ethical and professional values and qualities desired for physicians [7-9]. Some documents are directed to pediatricians [10-13] and discuss the methods for teaching ethical and professional values to undergraduates and residents in pediatrics [10,13].

However, there is still a gap between the ethical content taught in the universities and the ethical problems faced in clinical practice [14]. In addition to concerns about the adequacy of the formal curriculum, the influence of the hidden curriculum, that can lead students to learn and repeat the behavior observed in the supervisors and teachers, sometimes not adequate, has been highlighted for a long time [10,13,15,16]. This demands the identification of the ethical problems faced in all the learning settings and the seek for primacy in ethical behaviors.

Given that it is very important that medical students reflect about the best professional attitudes required to face the most common ethical problems that may arise in the different contexts where they attend children and adolescents, this study was developed to analyze the ethical problems experienced by physicians who have medical education and children and pediatric care responsibilities, and if those problems are associated to the workplace, their medical specialty and area of clinical practice.

## Methods

The study design had a mixed approach: cross-sectional, observational, descriptive and inferential and qualitative exploratory. The study population was composed of physicians who had teaching activities with undergraduate medical students from the *Universidade Federal de Santa Catarina *(Federal University of Santa Catarina), located in Florianópolis, capital city of Santa Catarina State-Southern Brazil) and/or residents and who attended children and adolescents in teaching hospitals or Basic Healthcare Units (BHUs). From the list provided by the management sectors of these institutions and from the university, the universe of 173 physicians was identified: 136 worked in hospitals and 37 in BHUs. The inclusion criterion included: concurrent role as a provider of children and adolescent health care and of medical education (undergraduation and/or residents). The exclusion criteria were: being a resident, being retired or licensed, not having direct contact with trainees in pediatrics and not working with child care.

To ensure that all the pediatric subspecialists working in the settings surveyed would be represented, the sample was selected by convenience. The estimated sample size of 72 participants was calculated using the Epi Info 2000 software and the following parameters: a total of 173 physicians, prevalence of 60% of ethical problems reports, relative risk of 3.0, test power of 80% (beta error type) and a 95% confidence interval (alpha error type). Initially, 110 physicians were invited to participate in the study; two declined, and 20 (16 from hospitals and 4 from BHUs) accepted to participate but did not complete the data collection instrument. Thus, the final convenience sample was composed of 88 physicians, 72 who worked in hospitals and 16 who worked in BHUs.

After approval of the study project by the Research Ethics Committee of the Joana de Gusmão Children's Hospital-Florianopolis, Brazil (Report 032/2008), data were collected by a self-applied, semi-structured questionnaire based on Taquette et al. [17], with three sections with the following aspects: 1. Closed-ended questions with socio-demographic and occupational variables: gender, marital status, religious belief, length of time working as a physician, medical specialty, pediatric area of activity, ethics/bioethics training, workplace; 2. Open-ended questions requesting the report of up to three situations experienced in the care of children and adolescents that represented an ethical dilemma, the feelings aroused in those situations, who or what helped and could have helped, what aids were used to the process of decision making and what was done; 3. Open-ended question requesting suggestions for strategies to best approach these situations. A pilot study was performed with 15 eligible participants.

The term *ethical dilemma *was used in the questionnaire, because it is the most used term in the medical field for the situations that the authors intended to study. Conceptually, *dilemma *corresponds to a situation in which only two choices are possible and only one of them can be correct [18]. As some situations reported by the participants did not involve dilemmas, to encompass all the situations reported, we opted to use in this study the term *ethical problem*, a more comprehensive concept, which involves situations for which we are not always able to identify solutions [18].

Data analysis: A thematic content analysis of the qualitative data was performed [19]. In the pre-analysis the qualitative data were passed to an individual card without the sociodemographic data to ensure the anonymity and the analysis was performed separately by both researchers by grouping the data into units of meaning and then categorizing them. In posterior meetings, the categories listed by each researcher were discussed and the definite categories were decided by consensus. Those categories were entered as categorical variables into a Microsoft Office Excel^© ^database (Microsoft Corporation, U.S.) along with the other variables in the questionnaire. In addition to descriptive analysis, the association between the frequency of each category of ethical problems reported (outcome) and the participant sociodemographic and occupational characteristic (independent variable) was tested using chi2 or Fischer Exact Test when appropriated. For the outcome "ethical problem category" a Poisson regression was performed, to analyze the prevalence ratios (PR) of the following exposure variables: medical specialty [i.e., pediatrics or family medicine (reference)], area of practice in pediatrics [i.e., pediatric subspecialty, general pediatrician or family physician (reference)] and workplace [i.e., hospital or primary care (reference)]. Because family physicians and other pediatricians who worked in BHUs did not report ethical problems related to end-of-life care, to estimate the PR of this outcome, only the variables general pediatrician versus subspecialist pediatrician were used. This analysis was adjusted for the following confounding variables: gender, age, marital status, religious belief, training in ethics and bioethics, and length of time working as a physician.

To ensure proportionality, the sample was weighed in relation to the frequency of general pediatricians, subspecialist pediatricians and family physicians in the universe of physicians with teaching activities with students from the *Universidade Federal de Santa Catarina *and children care practice in the 2 teaching hospitals and in the Basic Healthcare Units. For the statistical analysis, Stata 11.0 (StatCorp, College Station, TX, US) was used. A significance level of *p *< 0.05 was adopted.

## Results

The average age of the 88 participants was 44.1 years (CI: 42.2-46.1), the average length of time working as a physician was 19.6 years (CI: 17.6-21.5), the average time spent in daily care of patients was 6.8 hours (CI: 6.3-7.4) and that spent on medical students and residents education was 2.3 (CI: 1.8-2.7).

Among the 210 reports, five broad categories of ethical problems were identified. These ethical problems [with their frequencies, including absolute number (n), percentage (%) and 95% Confidence Interval (CI)] were related to:

a. ***Physician-patient relationships ***[n = 61 (29.0%, CI: 2.9-35.1)], which comprised difficult interactions with the patients and/or their families including issues such as:

- To ensure confidentiality, especially in adolescent care;

- To cope with difficult revelations (communication of bad news, disclosure of diagnosis, disagreement with diagnosis given by other physician);

- To cope with parents non-adherence to patients' treatment;

- To deal with difficult relationship with the patients' parents;

- To cope with unexpected reactions from family members;

- To manage parents beliefs;

- Conflicts involving the autonomy of parents and adolescents.

b. ***End-of-life care ***[n = 55 (26.2%, CI: 20.3-32.1)], which involved challenges and conflicts in terminal situations including issues such as:

- To take the decision to withdraw or whether to withhold or not advanced life support, nutritional support and resuscitation;

- To accept the decision of colleagues of admitting the patient in the Intensive Care Unit;

- To accept the decision of colleagues of prescribing futile therapies;

- To deal emotionally with the situation of patients without therapeutic perspectives;

- To diagnose brain death.

c. ***Health professionals conducts ***[n = 50 (23.8%, CI: 18.0-29.6)], which comprised disagreement with physicians or other health professionals conducts such as:

- To disagree with colleagues in the indication of procedures;

- To witness workplace inappropriate attitudes of colleagues in their relationship with patients and other colleagues;

- To disagree with inappropriate personal attitudes of physicians from other workplaces;

- To disagree with inappropriate patient relationship of physicians from other workplaces;

- To disagree with the breach of confidentiality, inappropriate use of medicines or inappropriate personal attitudes of other health professionals.

d. ***Socioeconomic issues and public health policy ***[n = 31 (14.8%, CI: 10.0-19.6)], which involved challenges concerning socioeconomic conditions and the public health care system that influence patient treatment, management and protection such as:

- To have to take decisions when the absence of inpatient beds threatens the lives of patients and surgeries are postponed;

- To cope with the social reality of patients, which imposes limits to the adequate management of care, resulting in lack of therapeutic success;

- To cope with the difficulty in referring patients to specialists;

- To cope with violence against children, including neglect;

- To experience problems in the workplace, among them, the lack of specialists, of equipments and of material;

- To cope with problems in the health care system that result in difficulties for patients to have access to more sophisticated diagnostic exams and to surgeries.

e. ***Pediatric Education Process ***[n = 13 (6.2%, CI: 2.9-9.5)], which comprised inadequate personal attitudes and interpersonal interactions in the academic environment including relationship between: student-teacher/supervisor, teacher-supervisor, teacher/supervisor-patient, student-patient, teachers-physicians of Basic Health Units such as:

- To witness an ethically reprehensible attitudes of the teachers;

- To witness medical undergraduate students disrespect for the university hierarchy;

- To experience problems such as the allowance by teacher/physician supervisor to residents to act when there is risk to the patient;

- To experience problems in the relationship professor/physician supervisor-patient, such as inadvertent exposure of patients and discussion of cases in corridors;

- To experience problems in the relationship between teachers/physician supervisors, such as public criticism and disrespect authorship in scientific publication;

- To witness problems in the personal attitudes of undergraduates and residents.

The distribution of the ethical problems reported according to the sociodemographic and occupational characteristics of the participants is presented in Table 1.

**Table 1:** Distribution of the ethical problems according sociodemographic and occupational characteristics of the participants.

		Ethical Problems related to*				
**Participants characteristics**	**n (%)**	**PPR** **(row%)**	**ELC** **(row%)**	**HPC** **(row%)**	**SEPHP** **(row%)**	**PEP** **(row%)**

***Gender***						

Male	38 (43.2)	50.0	36.8	39.5	26.3	5.3

Female	50 (56.8)	42.0	42.0	40.0	28.0	12.0

***Marital Status***						

Single	18 (20.4)	44.4	44.4	33.3	33.3	16.7

Married	60 (68.2)	50.0	38.3	43.3	23.3	6.7

Divorced	08 (9.1)	25.0	37.5	37.5	37.5	12.5

Living with partner	02 (2.3)	-	50.0	-	50.0	-

***Religious belief***						

Yes	72 (81.8)	43.1	41.7	40.3	26.4	8.3

No	16 (18.2)	56.3	31.3	37.5	31.3	12.5

***Specialty Area***						

General pediatrics	16 (18.2)	31.3	31.3^‡^	62.5	18.8	6.3

Pediatric subspecialty	58 (65.9)	46.6	51.7^‡^	36.2	24.1	10.3

Family Medicine	14 (15.9)	57.1	0.0^‡^	28.6	50.0^†^	7.1

***Bioethics/bioethics training***						

Yes	37 (42.0)	54.1	37.8	37.8	27.0	13.5

No	49 (55.7)	38.8	40.8	40.8	26.5	6.1

Non respondents	2 (2.3)	-	-	-	-	-

***Workplace***						

Teaching Hospital	72 (81.8)	41.7	48.6^#^	41.7	22.2^†^	9.7

Basic Health Unit	16 (18.2)	62.5	0.0^#^	31.3	50.0^†^	6.3

(Florianópolis-Brazil, 2010)

Notes: *PPR *Physician-Patient Relationship; *ELC *End of life Care; *HPC *Health Professional Conducts; *SEPHP *Socioeconomic issues and Public Health Policies; *PEP *Pediatric Education Process

*Values exceed 100% for the possibility of up to 3 reports by participant; the association between the category of ethical problem and participant characteristics was tested by Chi2 or Fisher exact when appropriated; ^† ^*p *< 0,05; ^‡ ^*p *< 0,01; ^# ^*p *< 0,001.

**Figure 1:**
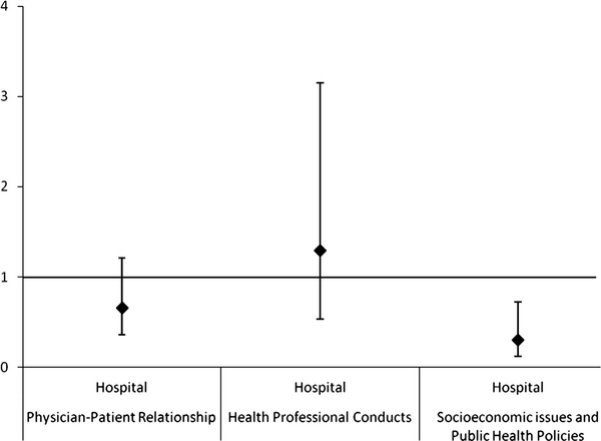
**Prevalence Ratio: work in hospitals compared to work in the Basic Health Units (exposure) and presence of at least one report in the category of ethical problem (outcome)**. Note: Two outcomes were omitted: End of Life Care (not reported by family physicians) and Pediatric Education Process (CI very broad).

**Figure 2:**
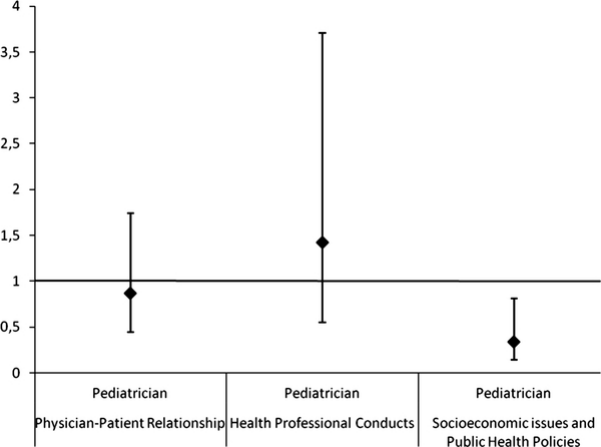
**Prevalence Ratio: clinical practice is as pediatrician compared to clinical practice as family doctor (exposure) of at least one report in the category of ethical problem (outcome)**. Note: Two outcomes were omitted: End of Life Care (not reported by family physicians) and Pediatric Education Process (CI very broad).

**Figure 3:**
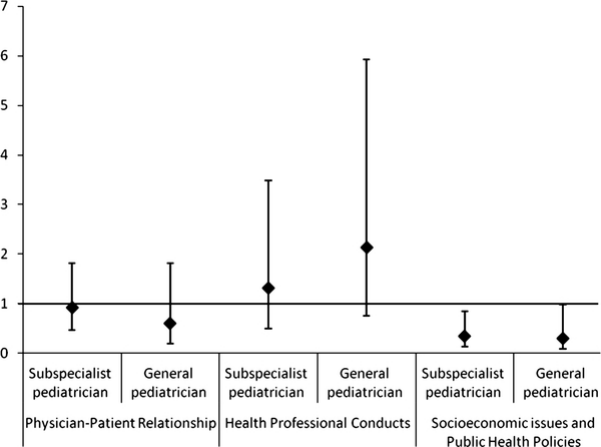
**Prevalence Ratio: medical specialty-general pediatrician or subspecialist pediatrician compared to family doctor (exposure) and presence of at least one report in the category of ethical problem (outcome)**. Note: Two outcomes were omitted: End of Life Care (not reported by family physicians) and Pediatric Education Process (CI very broad).

In Poisson regression, it was found that fewer ethical problems related to the SEPHP were reported among the participants who worked in hospitals when compared to those who worked in the Basic Health Units [PR = 0,3 (CI 95% 0,12-0,72)] (Figure 1), as well as among those whose clinical practice is as pediatrician (general and subspecialties in pediatrics) [PR = 0,34 (CI 95% 0,14-0,81)] when compared to clinical practice as family physicians (Figure 2). This association was maintained when medical specialties were compared: family physicians to subspecialists pediatricians and general pediatricians [PR = 0,3 (CI 95% 0,09-0,98) and PR = 0,35 (CI 95% 0,14-0,85)] (Figure 3). There was no statistical significance in the prevalence ratio of ethical problems related to PPR, HPC and PEP, when comparing workplaces, medical specialties and areas of clinical practice, which shows that the frequency of reports of these categories of ethical problems was similar among the participants. The category of ethical problems related to end of life was only reported by the participants who worked in Hospitals and no statistical significance was found in the prevalence ratio of this category when comparing general pediatricians to subspecialty pediatricians.

## Discussion

This study showed that the ethical problems experienced in the daily care of children and adolescents go beyond those usually described in the literature [20-22] and cover areas that should be planned for and addressed in the curricula for students of pediatrics. Some problems were more specific to certain workplaces, such as those related to end-of-life care situations occurring in hospitals and those related to socioeconomic issues and health policies occurring in primary care. This observation reinforces the conclusion of a meeting of experts in pediatric education [10] which was conducted in 2007. They concluded that the activities of pediatricians in their various work environments and subspecialties are sufficiently different to justify different training depending on the environment in which the professional is located. However, a high frequency of reports concerning interpersonal relationships was noted and was experienced equally in the health care and education settings. These relationships involve physicians, patients and families, the various professionals working in pediatric care, students, residents, teachers and supervisors.

The fact that many reports involved the physician-patient relationship reflects the importance that should be given to this subject in medical education. It is well established that this relationships should be of caring, built in the light of ethics and that it is strongly influenced by the moral values of those involved in this process, by the culture, the society and by the manner which the health care system is structured. To achieve the ideal standard of this interaction, the *American Academy of Pediatrics *(AAP) has established eight components of professionalism to teaching and assessment in pediatrics. Of these, six are directly related to the attitudes and values expected from the physician in relation to the patient (honesty and integrity, reliability and responsibility, respect for others, compassion and empathy, communication and collaboration, and altruism and defense) and the other two (self-improvement and self-awareness and knowledge of limits) relate to physician singular skills. These components must be worked on throughout medical education and on an ongoing process of continuing education after graduation [12].

Ethical problems involving conduct of health professionals also occurred in all surveyed environments. The situations included in this category, such as divergences in personal and professional conduct and difficulties in maintaining privacy, demonstrate the importance of developing negotiating skills and improving interactions with all participants in the health care network. Delany et al. [15] name as "allied health" in Pediatrics the professionals from many specialized health professions who work in the pediatric area in the health care team, attending children and adolescents with acute and chronic diseases or with disabilities. The relationship between physicians and these professionals may lead to ethical problems due to their differing perspectives of what constitutes the best interests of the child, which depend on what the authors call "disciplinary paradigms of care or operational philosophy." For these authors, it is necessary that the professional who attends the pediatric age group be aware of his role as moral agent, of his professional responsibility and of the impact of his decisions in the children and their families lives when he refers the patient to allied professionals [15].

Although the PEP ethical problems were reported by teachers and supervisors, the findings coincide with the finding of studies with students reports [21-25] which involved mainly disrespect when relating with patients, colleagues and students. The study reveal that in the education process it is essential an adequate communication between the parties and that it is expected that the teacher/supervisor be a role model and also that the student have appropriate ethical attitudes, especially a respectful way in the interaction with patients and teachers, and, for achieving this, educational actions are also needed.

The ethical problems related to end of life care were those more closely related to the impact of technological development in health, which require constant reflection of the ethical aspects. For this area, some of the important subjects in the teaching of Ethics in pediatrics should be emphasized such as the limits of prematurity, advanced life support in children with very limiting disabilities and severe malformations, do-not-resuscitate orders, therapeutic futility and palliative care, technology-dependent children and the use of off-label medications. Previous studies addressing these issues, which were developed in different settings, highlight the difficulties encountered by professionals working in hospitals, especially those in pediatric subspecialties who are entrusted with the care of critically ill children and adolescents [26-33]. They reinforce the need for physicians to have skills to cope with these situations so that their decision-making can achieve the patient's best interests.

The socioeconomic context and public health policies are complex and are an inseparable part of medical activity, as they are directly related to the medical work, particularly of those who attend pediatric patients, due to the the eco-dependency of the child. Problems of this scope are related to Social and Community pediatrics, which for almost a decade was considered by DeWitt [34] as the greatest challenge for the planning of educational activities, as it requires the inclusion of issues related to equity in child health and social justice. It is in the community context that the student has the opportunity to interact with the social determinants of health, to promote preventive action at different levels and to develop an interest in protecting children's rights [34,35]. In recent years, the relevance of teaching pediatrics in the community [34,36] has been recognized, and efforts have been made to change the predominantly hospital teaching model and insert the students in all levels of care. Decision-making in this context involves interdisciplinary team work, depends on political decisions and is often hampered by the need for changes in the political and social structure in which the child is placed. Pediatrics education must address issues of this nature and there is a need for faculty development to ensure adequate orientation of students at this level [37]. Also, pediatricians and family physicians can contribute positively to the encouragement, support and the establishment of effective partnerships with families [38], having active participation in health care teams. The AAP suggests that philosophies, principles and practices should be focused and targeted at health care in the family (family-oriented care), i.e., the family should be considered the unit of care and intervention. This approach make easier the understanding of the physician responsibilities, since the assessment of the emotional and social problems that affect the welfare of the child must always be included [38].

The generalizability of the findings of our study is limited, as the topic of ethics is influenced by socio-cultural characteristics and because there are regional differences in the characteristics of pediatric care and medical teaching. Other limitations may be related to the fact that the sample may be representative only of the environment where the research was conducted (Southern Brazil). Despite this fact, we expect that this study contributes as a basis for comparison with other cultures and regions and to the formulation of educational initiatives leading to the teaching of ethics and professionalism geared towards the practice of health care among children and adolescents. In this context, the ethical problems, experienced in different settings, reported in our study by pediatricians and family physicians who participate in the medical education process and attend children and adolescent could be used in the medical undergraduation, graduation and postgraduation curriculum and in faculty development programs as a means to raise critical reflection for and on action and promote ethical attitudes and professionalism.

## Conclusions

Some ethical problems that are experienced by physicians who treat children and adolescents are more specific to the workplace. Problems related to end-of-life care, such as those related to the decision to withdraw or withhold advanced life support, nutritional support and resuscitation, are more frequent in the hospital setting. Ethical problems associated with socioeconomic issues and public health policies that influence patient treatment, care and child protection are more frequent in Basic Health Units. Therefore, educational strategies to aid in the decision-making process and the ethical reflection on end-of-life care situations in pediatrics should be addressed among medical students and professionals to prepare them for pediatric practice in hospitals. The ethical problems related to socio-economic issues and public health policies need to be approached in pediatrics and adequately discussed, specially linked to the teaching of pediatric primary health care and to the community practice.

Others problems, however, are present in all the settings of pediatric care and learning and include ethical problems related to physician-patient relationships, health professional conducts and the pediatrics education process. So, the teaching of communication skills for effective physician-patient relationship and interdisciplinary team work, anchored in professionalism in pediatrics, rooted on ethical attitudes, should be planned and provided along all the years of medical course, in academic activities at all the levels of health and should be present in all the learning environments.

Given the importance and frequency of the ethical problems reported by the physician in different settings of pediatric clinical practice, they could be used in the training of students, physicians and faculty as a means to raise critical reflection for and on action and promote ethical attitudes and professionalism.

## Competing interests

The authors declare that they have no competing interests.

## Authors' contributions

JMG conceived of the study, participated in its design, data collection and analysis and drafted the manuscript. SG conceived of the study, participated in its design, data analysis, as well as in the critical review of the manuscript. All authors read and approved the final manuscript.

## Pre-publication history

The pre-publication history for this paper can be accessed here:

http://www.biomedcentral.com/1472-6939/13/2/prepub
